# Radar Data Integrity Verification Using 2D QIM-Based Data Hiding

**DOI:** 10.3390/s20195530

**Published:** 2020-09-27

**Authors:** Raghu Changalvala, Brandon Fedoruk, Hafiz Malik

**Affiliations:** Department of Electrical and Computer Engineering, University of Michigan-Dearborn, Dearborn, MI 48128, USA; bfedoruk@umich.edu (B.F.); hafiz@umich.edu (H.M.)

**Keywords:** radar objects, 2D QIM, sensor fusion, watermarking, CAN, sensor data integrity, kalman filter

## Abstract

The modern-day vehicle is evolved in a cyber-physical system with internal networks (controller area network (CAN), Ethernet, etc.) connecting hundreds of micro-controllers. From the traditional core vehicle functions, such as vehicle controls, infotainment, and power-train management, to the latest developments, such as advanced driver assistance systems (ADAS) and automated driving features, each one of them uses CAN as their communication network backbone. Automated driving and ADAS features rely on data transferred over the CAN network from multiple sensors mounted on the vehicle. Verifying the integrity of the sensor data is essential for the safety and security of occupants and the proper functionality of these applications. Though the CAN interface ensures reliable data transfer, it lacks basic security features, including message authentication, which makes it vulnerable to a wide array of attacks, including spoofing, replay, DoS, etc. Using traditional cryptography-based methods to verify the integrity of data transmitted over CAN interfaces is expected to increase the computational complexity, latency, and overall cost of the system. In this paper, we propose a light-weight alternative to verify the sensor data’s integrity for vehicle applications that use CAN networks for data transfers. To this end, a framework for 2-dimensional quantization index modulation (2D QIM)-based data hiding is proposed to achieve this goal. Using a typical radar sensor data transmission scenario in an autonomous vehicle application, we analyzed the performance of the proposed framework regarding detecting and localizing the sensor data tampering. The effects of embedding-induced distortion on the applications using the radar data were studied through a sensor fusion algorithm. It was observed that the proposed framework offers the much-needed data integrity verification without compromising on the quality of sensor fusion data and is implemented with low overall design complexity. This proposed framework can also be used on any physical network interface other than CAN, and it offers traceability to in-vehicle data beyond the scope of the in-vehicle applications.

## 1. Introduction

In the last 20 years, the automotive industry has seen rapid growth in the usage of electronic control units (ECUs) to implement various technology features, such as dynamic vehicle control, infotainment, and ADAS safety features. These technologically-enhanced user experience and safety features are heavily dependent on the sensors and advanced communication networks integrated into the vehicles. An autonomous vehicle (AV), in particular, depends heavily on sensors and data transmission networks to implement the highly automated driving functions [1]. Autonomous vehicles rely entirely on sensors to estimate their surroundings, to detect and react to the obstacles. To achieve a sustainable society of automotive engineers (SAE), level 2 automation and above [2], a typical vehicle is equipped with multiple sets of sensors, including cameras, LiDARs, radars, etc. To get perspective, the GM Cruise AV is equipped with 5 LiDARs, 16 cameras, and 21 radar sensors [3]. The sensor data flows through the vehicle network to reach the centralized data processing unit called the advanced driver assistance system (ADAS) module or the vehicle’s brain from the sensors mounted on the vehicle. An autonomous vehicle internal communication network is widespread and relies on multiple physical interfaces, including the controller area network (CAN), CAN-flexible data rate (CAN-FD), Ethernet, local interconnect network (LIN), FlexRay, etc. The vehicle internal network topology, which consists of ECUs, gateways that forward data from one interface to other, and the connectivity to the external world over cellular and other wireless interfaces make the modern vehicle a cyber-physical system vulnerable to cyber attacks [4]. In a given vehicle with autonomy levels higher than SAE level 2 (semi-autonomous mode), a cyber-attack on the sensor data could lead to life-threatening accidents, as the driver could be disengaged partially or completely from the system [5]. Hence, securing sensor data transmissions is a very critical component for the safe functioning of an autonomous vehicle [6].

Autonomous vehicle sensors are broadly divided into smart and raw categories. The raw sensors—specifically, the ones that have high data rates, such as the camera and LiDAR—use an Ethernet interface for raw data transmissions to the ADAS unit, whereas smart sensors that can detect and track objects internally send the tracked object list to the vehicle over limited bandwidth interfaces. The majority of sensor sets available for use in autonomous vehicles are sold as smart sensors. These sensor units can sense and track objects internally and send out the tracked data over a deterministic and fault-tolerant interface such as CAN/CAN-FD. The data from these smart sensors are fused by the vehicle ADAS unit to determine the final list of surrounding objects [7].

Since most of the smart sensors use CAN or CAN-FD interfaces for data communication, a vulnerability assessment to cyber attacks is prudent for these network types. The CAN interface is widely used in the automotive industry due to robust and fault tolerant design; however, CAN lacks inherent security to protect against different network attacks. The shortcomings in CAN protocol such as the broadcast message format, clear data transfers, and lack of mechanisms to establish data authentication and confidentiality, expose CAN networks to masquerade attacks, replay attacks, and additional exploits [8]. The number of viable attack vectors on CAN networks has been demonstrated on numerous occasions within automotive security research [9]. To mitigate known vulnerabilities in CAN, several methods, such as payload encryption, frame ID-based filtering [10], and message authentication code (MAC) calculated based on the payload data have been developed. A transport layer security architecture can be built over CAN that can combine both cryptography and MAC to ensure data integrity and authenticity. As more vehicle manufacturers adopt Automotive Open System Architecture (AUTOSAR)-based platforms for vehicle development, Secure Onboard Communication (SecOC) is gaining traction, which is again another MAC-based authentication protocol for individual protocol data units (PDUs) [11]. When it comes to adopting these traditional cryptography and MAC-based security mechanisms in autonomous vehicles, many practical issues, such as key management, freshness value handling, and the recovery strategy or how to deal with the failed authentications, need to be taken into consideration. Additionally, the bandwidth and payload length restrictions of the CAN network make it impossible to implement these methods. The legacy systems need to upgrade to CAN-FD that shares the same architecture as CAN but provides more bandwidth due to flexible data rates [12]. Resolving such practical issues increases the development and maintenance costs of the product, and the overall complexity.

To deal with such implementation level and practical shortcomings of the traditional data integrity verification methods, we introduce a new data hiding-based watermarking approach. This approach solves the problem of the data integrity verification in resource-constrained and real-time applications with simple algorithms that do not tax the system with high computational complexity and at the same time do not increase the bandwidth requirements of the interface, as no additional data are added to the payload. In this method, the watermark is embedded into the sensor using a light-weight software algorithm, which is easy to implement. The concept of using data hiding techniques for sensor data verification was introduced in [13] for raw sensor data used in a centralized autonomous driving architecture [14]. In this paper, we analyze a different modality; we consider a smart radar sensor that produces the processed data. The proposed watermarking method was implemented on the processed radar data and its effects on the outcome of a sensor fusion algorithm were verified.

### 1.1. Watermarking Advantages

In this digital age, with the growing trend of multimedia information exchange, the need to ensure data confidentiality and integrity is increasing. Data transfers over different networks are vulnerable to various attacks such as privacy infringement, content stealing, and tampering. The concept of the network information security deals with securing the data transmissions, storage, and processing from data leakage, theft, tampering, and deletion [15]. To ensure the network information security, traditionally cryptography based methods were used. Cryptography ensures communication privacy, data confidentiality, and authentication by encrypting the data using different key sharing mechanisms. Though cryptography can be used to solve the information security issues, there are some disadvantages. Encryption can draw an attacker’s attention towards sensitive information and motivate them to crack it. Once the attackers break the encryption, they have complete access to the data. Even if the attacker fails to crack the encryption, he can slightly modify the data to make the entire transaction invalid [15]. Data hiding-based watermarking techniques started gaining attention over the past two decades, particularly in the areas that require prevention of unauthorized access to confidential information. These methods do not reveal their existence in the data, thereby providing more protection than cryptography in some applications. Data hiding methods differ from traditional cryptography in their purpose. The main objective of data hiding is not to restrict normal access to the data but to ensure that the embedded secret information is not violated or discovered [15]. This embedded message can be used to track the data forgery through different data transactions and also to localize it. The cryptography methods are usually complex and computation resource hungry; hence they cannot be applied everywhere. In the edge computing devices such as AVs, where the computation resources are a big constraint, cryptography methods cannot be applied. There are also other significant drawbacks of using cryptography methods in edge systems, such as key management burden, network bandwidth issues, and export restrictions on the products using specific cryptography methods. These complexities increase both the production and maintenance costs of the features and products. In an AV, consider a scenario where a couple of satellite radars are connected over a CAN network to a decision-making unit that does the sensor data fusion to implement an ADAS feature. To implement sensor data verification using traditional cryptography techniques requires modifying each smart radar sensor to include a hardware trust anchor to store the keys and accelerators to verify the signatures. This increases the radar product cost and any MAC-based security mechanism will also increase the payload overhead, thereby requiring a different interface than CAN that supports higher bandwidth, which again is not feasible in legacy systems.

**Figure 1:**
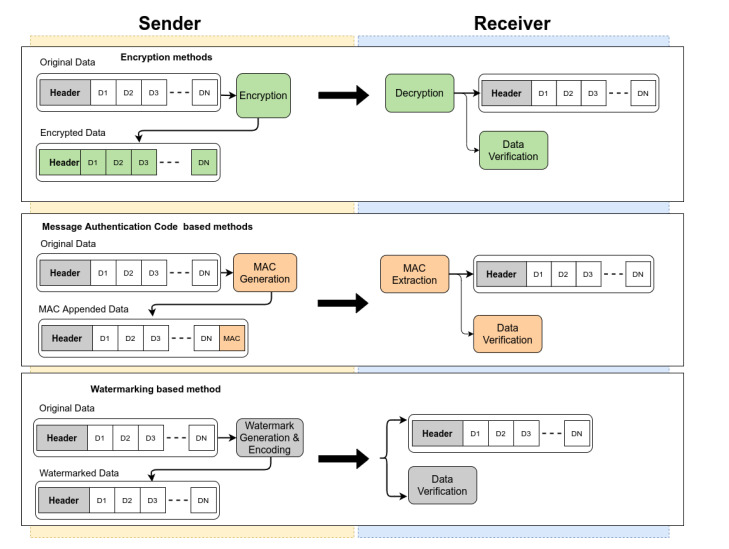
Comparison of different methods to achieve sensor data integrity.

In comparison to the conventional cryptography, watermarking methods are computationally less complex and less resource hungry. Data modified by the watermarking algorithms can be directly used by the end application without having to modify or to clean it before usage. This comes as an advantage for applications that rely on real-time data processing. In Figure 1, different methods that can be used to protect and verify the data integrity in an AV are compared. In a sender–receiver scenario, to protect the data integrity, one can use the encryption, MAC embedding, or watermarking. In both encryption and MAC-based methods on the receiver end, applications cannot use data until the it is decrypted or the MAC is removed. This additional computational step can become a bottleneck in real-time applications. In MAC-based methods, the data payload increases dramatically based on the interface bandwidth. For CAN interface in particular, this method is not recommended as it could double bandwidth requirements [12,16]. Data hiding based watermarking works directly on the host data by perturbing the data by a negligible amount, thereby eliminating the need to increase the data payload and network bandwidth. As shown in Figure 1, all the three methods have a data integrity verification step in common that can be separated out as an independent process and run in parallel to the algorithms that process the data. Again, in the case of watermarking, this data verification step does not require much in the way of computational resources, unlike cryptography.

Watermarking also provides the much-needed data traceability. The security offered by watermarking does not stop at the application level. Watermarked data provide security beyond the autonomous driving application since the data cannot be stripped of any additional payload. The embedded watermark stays with the data until the information is used by the consumer application and beyond, which is not the case with the traditional cryptography techniques that end at the transport layer. Applications acting as pass-through sections to the data, such as an onboard data recorder and secure logging mechanisms that push the sensor data to cloud for analytics, CAN benefit from the watermarking of the data. When data get exchanged from one entity to another and security keys are shared, end-to-end encryption does not help in identifying the leakage point, whereas watermarking can. These advantages make data hiding-based watermarking techniques a better choice over the cryptography in many edge computing applications such as sensor data integrity verification in autonomous vehicles.

### 1.2. Principal Contribution

Automotive sensor networks have two constraints.

- **Limited bandwidth**: The communication interface from the sensor to the data processing unit is bandwidth limited in automotive applications. Most sensors use a traditional CAN interface with an 8-byte payload, that restricts the usage of traditional cryptographic methods, securing the sensor data [16]. An enhanced version of CAN called CAN-FD is introduced to increase bandwidth and payload to up-to 64 bytes. CAN-FD allows AUTOSAR secure onboard communication protocol (SecOC) implementation on the network. Apart from issues, such as key management and time synchronization, the SecOC requires the transmission of a message authentication code (MAC), which can take up to 8 bytes of the payload space. Bandwidth becomes a constraint even in high throughput interfaces such as CAN-FD. Consider a scenario where multiple sensors are connected to the same network, it takes no time to saturate the bus by adding an additional sensor to the network or by increasing the message frequency or data payload of each sensor. Legacy networks often run into these bandwidth issues given the increased data demand on the sensors to build high-resolution perception layer to support autonomous driving features.
- **Real-time data integrity verification**: Autonomous vehicle applications often require the sensor data to be processed in real-time. This constraint makes it difficult to use traditional data security methods based on cryptography as they require an additional step of decryption before data becomes useful.

**Figure 2:**
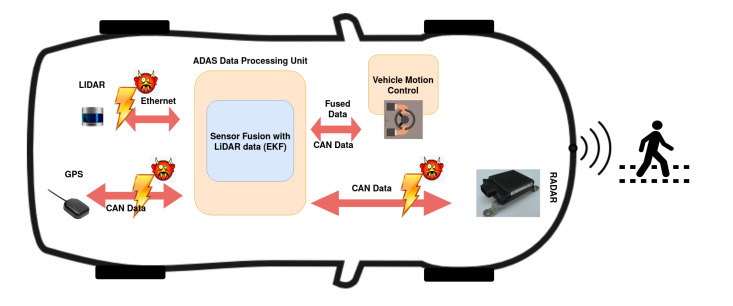
Block-diagram of problem statement.

With these constraints and given the lack of cyber-security mechanisms in CAN/CAN-FD, the in-vehicle network can get hacked at the data interfaces or transport layer, as shown in Figure 2. In this paper, we propose a watermarking solution that can work well under these constraints and yet help verify the integrity of the sensor data. The traditional watermarking methods are vulnerable to watermark estimation attack. To address it, we propose to introduce freshness in the watermark generation process based on the data available on the in-vehicle network such as the GPS timestamp to generate a watermark to be embedded into the sensor data that need to be secured. Sharing the watermark generation algorithm between the sensor and the receiver eliminates the need to exchange the watermarking scheme over any secure channel. Another aspect to consider while using the data hiding based watermarking techniques is the embedding-induced distortion and one of the significant contributions of this research is to provide an analysis on embedded-induced distortion and its impacts on sensor data and downstream fusion algorithms.

## 2. Related Work

Digital watermarking methods have a significant advantage over the traditional cryptography methods when it comes to the resource-constrained systems where the interface bandwidth or the computation resources are a bottleneck. State-of-the-art research in watermarking based techniques to secure resource-constrained systems leads us to wireless sensor networks (WSNs). WSNs have similar interface bandwidth constraints to those of legacy automotive networks. The dynamic nature of these networks makes them susceptible to adversary attacks, such as data tampering, forgery, selective forwarding, replay, and transfer delay. Hence, in this area, significant research has been done to secure data transfers using watermarking.

Using watermarking for sensor integrity checks in WSN started in 2003 with the Feng et al. [17] proposal of embedding cryptographically encoded signatures into the data payload. In [18], Ibrahim and Hussam introduced a fragile watermark technique for securing the wireless sensor networks from insertion, deletion, and replay attacks. In their proposed FWC-D method, they generated a serial number to attach each group of transmissions to help receivers in determining the insertions and deletions. The watermark is generated using a simple hash function. In [19], Ibaida et al. introduced watermarking methods to include patient information in the health data. Their approach is designed to preserve the main features of the ECG, even after embedding the patient metadata. A basic variation of QIM called the least significant bit (LSB) modification method, wherein the least significant bits of the linearly transformed host signal samples are replaced with the bitstream to be embedded, is evaluated in this work. In [20], a single bit watermark was generated using a simple XOR operation for a homogeneous sensor network. Sun et al. [21] proposed a watermark generating mechanism based on a one-way hash function. The watermark calculated for each data byte is aggregated using the XOR function and embedded at a predefined redundant space, making it a simple strategy that provides end-to-end integrity. Lalem [22] proposed a linear interpolation based watermarking method. It does not generate additional data for watermarks, but the usage if a fixed parameter for all nodes is a security vulnerability. In [23], the watermark was generated based on a key and length known to both source and sink. Additionally, to reduce the computation complexity, it is assumed that the data have header information and is time-synchronized. In other words, it is expected that all types of data in the one working cycle use the same collected time. Watermark bits are randomly placed (using the random generator) in the data header and then extracted and compared to find the integrity. In [24], Alromih et al. proposed a Randomized watermarking filtering scheme (RWFS) for IoT applications. The watermark is randomly embedded into the encrypted data using a pseudo-random generator. Random generator parameters are determined by the cluster from which the packet is generated. The embedded watermark is retrieved and compared to the generated watermark over a shared key. This method provides end-to-end data confidentiality due to encryption and data integrity from replay, injection, and modification attacks due to the watermarking.

In [25], Kanchan et al. discussed the concept of insider attacks on the LiDAR point cloud. Multiple approaches that exploit the resolution and occlusion consistency between the tampered and clean data frames are proposed. In [13], Raghu and Hafiz introduced a watermarking-based approach to secure raw LiDAR sensor data in autonomous vehicles.

## 3. System and Attack Model

**Figure 3:**
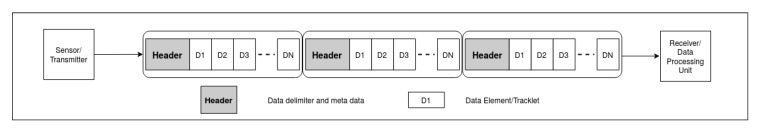
Radar data stream.

In the system model, we assume that a centralized ADAS unit makes autonomous driving decisions using the data fed by satellite sensors over the vehicle network, as shown in Figure 2. The ADAS unit fuses incoming data and performs the necessary information extraction from the object detection lists. This processed information is further used to build autonomous vehicle features such as perception and localization [13]. We assume that both the sensors or data origin, and the sink or the central data processing ADAS module are clean. The data input into the system via sensors is authentic. The attacks are launched during the data transmission from the sensor to the ADAS module over the vehicle network, as shown in Figure 2. This threat model is more attractive to attackers as the impact factor is high. The damage that can be done by continuously faking or deleting the sensor information as an insider attack is high for autonomous vehicle applications. The system requires a GPS receiver on the vehicle network transmitting timestamp data periodically, and the sensors, along with the ADAS unit, have access to this GPS data. The data structure shown in Figure 3 is assumed for the radar sensor data. Each dataset starts with a header delimiter that contains information such as the number of tracked objects, unique data identifiers, etc. The header is followed by the stream of data elements themselves that can contain multiple fields based on the capability of each radar sensor unit, but for simplicity, we assume the minimum content such as the tracked object Cartesian coordinates (x,y). This position information is used to embed the watermark. The proposed QIM-based watermark embedding method works directly on the data; hence it is important to verify that for a given application, the embedding-induced distortion does not affect the output of the application consuming the data. In this paper, we use a sensor fusion algorithm called the extended Kalman filter (EKF) that is widely used in autonomous vehicle applications to analyze the embedding-induced distortion. The sensor fusion algorithm takes in the radar and LiDAR sensor data and outputs the predicted position of the tracked object.

### 3.1. Sensor Fusion Data Model

**Figure 4:**
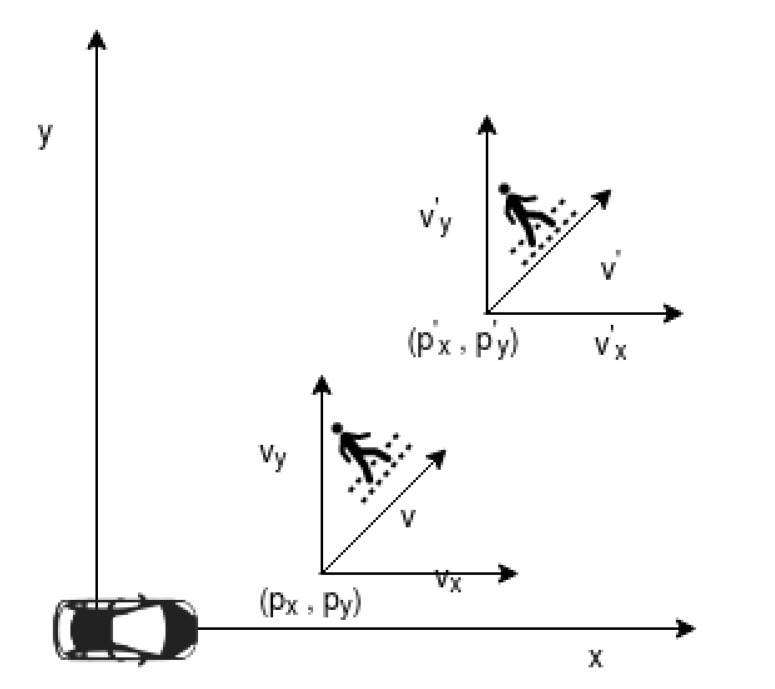
State vector for pedestrian motion.

The dataset used to test the proposed framework was generated using the reference code provided by Mercedes Benz autonomous driving utility [26]. The data generation scenario was to predict the path taken by a pedestrian walking in front of the vehicle using the onboard sensors, LiDAR and radar, as shown in Figure 4. The dataset contained sensor measurements of location and velocity of the pedestrian. The radar sensor measurements are represented in polar coordinates as ρ, ϕ, $\dot{\rho }$, where ρ is the radial measured distance to the target, ϕ is the measured lateral angle to the target, and $\dot{\rho }$ is the rate of change of ρ that results in radial velocity. The Radar measurements are converted from polar to Cartesian coordinates using the following equations.

(1) $$\begin{array}{c} x=\rho *cos(\phi )\\ y=\rho *sin(\phi ) \end{array}$$

The LiDAR measurements are represented as position coordinates (x,y). Along with these measurements, the dataset also contains the GPS timestamps for when the data were collected. The time delta between sensor measurements was set to δt=50ms. At each time sample δt, the ground truth values for the pedestrian position and velocity for each sensor $\left(p_{x},p_{y},v_{x},v_{y}\right)$ were calculated based on a constant velocity motion model. The motion model considered for data generation was a 2D bicycle model, and yaw-rate was assumed to be zero, represented by following equations:

(2) $$\begin{array}{cc} \; & \dot{\theta }=0\\ \; & x^{{}^{\prime }}=x+v\cdot \delta t\cdot cos\left(\theta \right)\\ \; & y^{{}^{\prime }}=y+v\cdot \delta t\cdot cos\left(\theta \right) \end{array}$$

where *v* is the target velocity, δt is the elapsed time, and θ is the yaw; (x,y) and $\left(x^{{}^{\prime }},y^{{}^{\prime }}\right)$ are the initial and final position values respectively. From those ground truth values, the measurement values at each time step were obtained by adding uncertainty in the form of Gaussian noise of configurable variance. The measured values are represented as

(3) $$m_{t_{k}}=g_{t_{k}}+\epsilon$$

where $m_{t_{k}}$ and $g_{t_{k}}$, represent the measurement and ground truth values respectively at time $t_{k}$, and ϵ is the measurement error represented by an independent and identical distribution (i.i.d.) Gaussian noise with zero mean and covariance matrix R>0, i.e., ϵ=N(0,R).

### 3.2. Watermarking Data Model

The watermarking model that fits the sensor data integrity verification problem is the side information based blind decoding method. In this model, the detector is unaware of the original host signal. Still, the auxiliary information such as the encoding message sequence or the key is used to accurately verify the data integrity. The host signal $c_{0}$ in this case, CAN be considered as a member of a set of *np*-dimensional vectors, $c\in \{c_{0},\cdots ,c_{n}\}$, where *n* is signal length. The dimensionality of the signal depends on the application. Considering the example of position data from a radar sensor, which is represented by a two-dimensional vector [x,y], the dimensionality *p* becomes 2. In data hiding-based methods, the host signal $c_{0}$ is transformed to a nearby value using simple techniques such as LSB modification. Using similar watermarking methods, numerous values are generated in the data space that is perceptually indistinguishable from the original data. Imagine a region around the $c_{0}$ in which every vector corresponds to the radar position data that is indistinguishable from the original position. This region is called the region of acceptable fidelity [27].

Once the embedder creates this dataset of similar position vectors, based on the input message *m*, the detector operates in the detection boundary. The detection region for a given message *m* and watermark key *k* is defined as the set of position vectors in the data-space that can be used to extract the embedded message. This region is defined by the threshold on a similarity measure, such as a linear correlation between the detector’s input and the original message *m*. During embedding, the message *m* is message mapped onto the pattern of the same type and dimensions of the host signal, and $w_{r}$ is one such reference pattern with possible dependence on a key *k*. The detection algorithm computes a linear correlation between the received signal and the reference pattern $w_{r}$; this is equivalent to the orthogonal projection of the received signal $c_{wn}$ onto the reference pattern $w_{r}$. Let, $c_{1}$ and $c_{2}$ be two p-dimensional vectors in the dataset, and if we set a limit $\tau_{mse}$ on the function that measures the perceptual distance or variation between the two vectors, such as a mean square error (MSE) measure, the region of acceptable fidelity is an n-dimensional sphere centered around the host signal $c_{o}$ with a radius defined as $\sqrt{n\tau_{mse}}$. The measure of successful embedding of a watermark is whether the watermark falls in the intersection of the acceptable fidelity and detection regions. Data hiding techniques perturb the host signal from generating a similar signal that falls within this intersection region, thereby making sure that the embedding-induced distortion is minimal and the embedded message is recovered with integrity. If the distortion exceeds certain threshold, it damages the watermark, making it feasible to detect and locate the data that got distorted. This behavior of the fragile-watermarking scheme QIM is used in this paper to detect and localize the data tampering.

## 4. Proposed Framework

**Figure 5:**
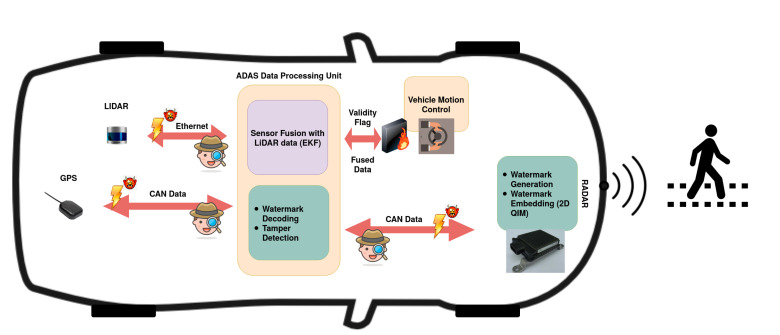
Block-diagram of proposed method.

In our proposed sensor data integrity verification framework, we assume the following. First, the sensor and the ADAS processing unit share the watermark generation algorithm. Second, the ADAS processing unit collects detection lists from different sensors, in this case, LiDAR and radar, and generates a fused list of detection. The proposed framework, as shown in Figure 5 is divided into three parts.

- Watermark generation.
- Watermark embedding.
- Watermark decoding.

The watermark generation is done in the sensor using the GPS timestamp information. This binary sequence of message $m_{e}=f\left(t_{gps}\right)$ is embedded into the position data of the object detection list from the sensor using 2D QIM data hiding method. This watermarked data are transmitted to the ADAS unit over an in-vehicle communication network, such as CAN. The ADAS unit receives the watermarked data along with the timestamp from the GPS sensor. These data are given as an input to the sensor fusion algorithm and to the integrity verification algorithm. The integrity verification algorithm that runs in parallel to sensor fusion generates the embedded message sequence $m_{e}$ using the same method as the sensor. Additionally, the embedded message sequence from the received frames $m_{d}$ is extracted using the decoding process. The decoded message sequence is compared against the embedded message sequence to detect and localize the modified data across different attack vectors, as explained in Section 5. When data tampering is detected, a validity flag is set to qualify the fused object list. This is represented as a fire-wall between the ADAS unit and the motion control unit in Figure 5, which prevents the tampered data from controlling the vehicle. The data integrity verification mechanism is portrayed as a silent spy in Figure 5. It acts in the background unnoticed by the attacker to detect and localize the tampering and alerts the vehicle control algorithm about the integrity of the data.

### 4.1. Watermark Generation

**Figure 6:**
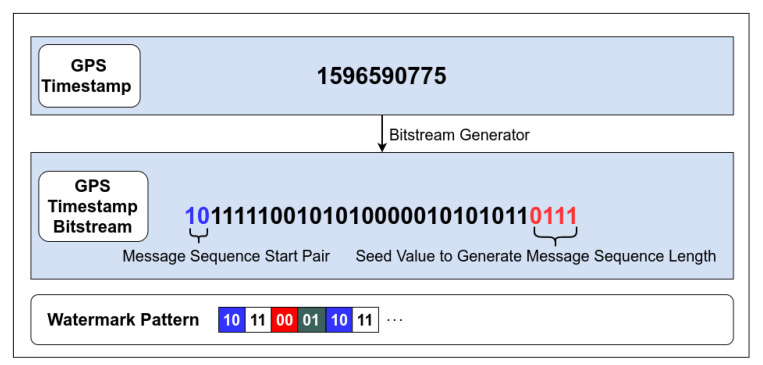
Time-stamp conversion.

A core component of our integrity verification network is our watermark generation technique. Our proposed model leverages timestamp from GPS sensor data to generate a binary sequence that will be embedded into subsequent radar data frames downstream. The diagram in Figure 6 represents a visual depiction of a GPS sensor timestamp converted into a bitstream. Assuming an architecture supporting little endianness, the two least significant bits (LSB) and the most significant bit (MSB) nibble are parsed and stored in a secured buffer, to be utilized by the sequence generator described in Algorithm 1. The LSB pair stored is used to determine the starting bit pair for the generated sequence. This adds a level of obfuscation to the generated sequence by changing the starting bit pair of the binary sequence to be embedded in the available data elements. In addition, the MSB nibble is converted to its decimal representation and utilized as a seed value to generate a random number to fall within the theoretical maximum for potential data elements generated in one message payload.
**Algorithm 1:** Watermark sequence generator.
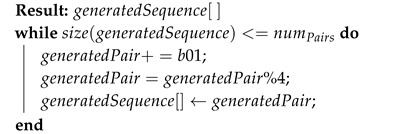


The sequence generator represented by Algorithm 1 will utilize the previously gathered information from the GPS time stamp to generate a deterministic sequence. This process involves taking the range limited random number *x*, generated from the seed value of the MSB nibble where: $num_{Pairs}=\left\lfloor x\right\rfloor$ to determine the length of the sequence. A two-bit value is then incremented and appended to the generated sequence buffer. The proposed 2D QIM embedding method allows for a message sequence of integer values in range $\left(0\leq m_{val}<4\right)$, hence the generated two bit value is modulated by 4, to keep it within the allowed range. The desired length of the sequence is, by design, dependent on the seed value calculated from parsing the GPS timestamp. If the sequence is shorter than the amount of data elements in the message payload, the generated sequence will be reused. The randomness in this generated message sequence comes in the form of the start pattern and the length of the message sequence both of which can be recreated by the receiver using the same GPS timestamp message.

### 4.2. Watermark Embedding

We use a 2D QIM-based data hiding method for watermark embedding. QIM is a non-linear, data hiding-based, semi-fragile watermarking method that is widely used in digital forensics and steganography [28]. In QIM a host signal $S=\{s_{1},s_{2},\cdots ,s_{N}\}$ is quantized based on the embedded message symbols $M=\{m_{1},m_{2},\cdots ,m_{N}\}$. If we consider a simple implementation of a binary QIM, where $m_{i}\in \left\{0,1\right\}$, the modified or watermarked host signal $S_{w}$ can be represented as:

(4) $$S_{w}=q_{m_{i}}\left(s_{i},\Delta \right),\;\mathrm{where}\;i=1,2,\cdots ,N$$

where, $q_{m_{i}}\left(\cdot \right)$ denotes a uniform quantizer. With a quantization step-size Δ and a perturbation of Δ/2, this uniform quantizer is represented as:

(5) $$q_{m_{i}}\left(s_{i},\Delta \right)=\mathrm{round}\left(\frac{s_{i}}{\Delta }\right)\cdot \Delta \pm m_{i}\cdot \Delta /2$$

It can observed from Equations (4) and (5) that the host signal gets modified after the data embedding and the distortion level is proportional to the perturbation. This feature provides the flexibility to select a distortion level that works for a particular end application. This motivated us to select QIM over other available watermarking methods.

**Figure 7:**
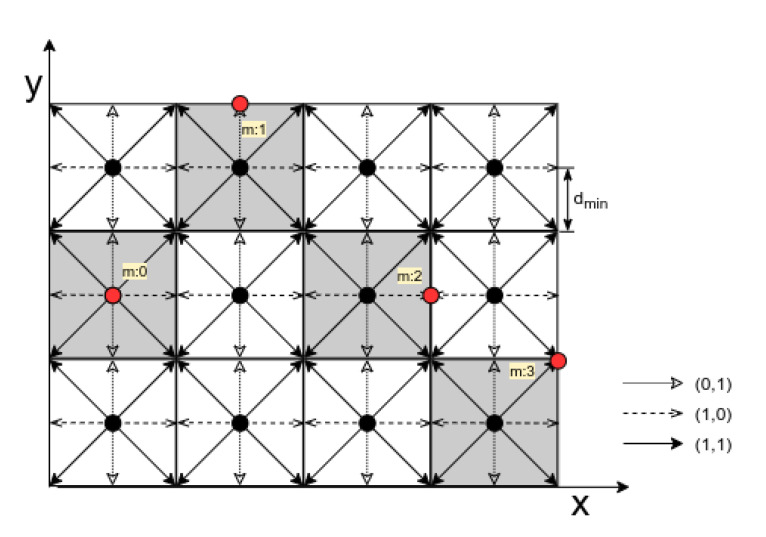
2D QIM scheme.

In QIM, the quantization operation uses a unique set of quantizers that result in a reconstruction grid [29]. The dimensionality of the reconstruction grid depends on the message symbol size. If a message has an *n*-dimensional symbol, it results in a $\log_{2}\left(n\right)$-dimensional reconstruction grid. For example, a binary message m∈{0,1}, where n=2 results in a 1D reconstruction grid. If we extend this concept to a two dimensional dataset such as radar data position vector (x,y), a four dimensional message symbol m∈{0,1,2,3} can be used to hide data resulting in a 2D reconstruction grid. Each corner of this grid can be considered as a reconstruction point in recovering the embedded message. In Figure 7 a sample dataset is depicted with different embedded message symbols. After the initial quantization, the position data points that fall within the highlighted polygons are represented by the center of the regular polygon or the black dot $c_{k}=\left\{x_{k},y_{k}\right\}$. In 2D QIM, based on the embedded message symbol $m_{k}=\left\{m_{xk},m_{yk}\right\}$, this center point is moved to one of the eight fixed locations on the boundary of the polygon as represented by the red dot. The minimum value of the separation distance between the reconstruction points $d_{min}$, determines the resilience of the framework to the channel noise. This is a configurable parameter in QIM-based watermarking that comes as an advantage when trying to adapt this framework to different end applications. Another advantage being the host-signal interference rejection because of the non-intersecting reconstruction points [30]. The resulting watermarked signal $s_{w}$ for a 2D QIM can be represented as:

(6) $$s_{w}\left(s_{c_{k}},m_{k}\right)=q_{m_{k}}\left(s_{c_{k}},\Delta \right)$$

where $q_{m_{k}}\left(\cdot \right)$, denotes 2D QIM quantizer which is expressed as:

(7) $$q_{m_{k}}\left(c_{k},\Delta \right)=\mathrm{round}\left(\frac{c_{k}}{\Delta }\right)\cdot \Delta \pm \frac{\Delta }{2}\cdot m_{k}$$

**Figure 8:**
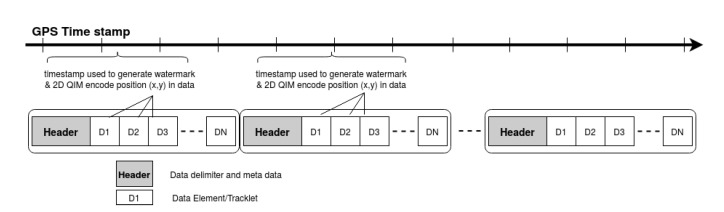
Proposed framework and 2D QIM embedding process.

In the proposed framework, a simple algorithm parses through the generated message symbols and applies the corresponding quantizer to the radar position data as per Equation (7). The sender uses the GPS timestamp from a pre-defined interval and is based on the procedure explained in Section 4.1; the watermark is generated and embedded into the data elements of the radar data, as shown in Figure 8. The modified data are transmitted over the selected data transfer interface, for instance, CAN/CAN-FD, with additional meta-data included in the header that acts as a delimiter to the data-elements. The watermark stays with the data irrespective of the data-link or transport protocols used to send the data to the receiver. Additionally, since there is no additional data added in the form of MAC, the interface bandwidth requirements remain the same.

### 4.3. Watermark Decoding

The radar detection object list data received by the ADAS unit can be directly used by the sensor fusion algorithm. Tamper detection and localization algorithms can run in parallel. This is a significant advantage of the watermarking method over any other encryption-based methods. The decoding algorithm works similarly to the embedding. Each received position value is quantized using all the different quantizers used for embedding to generate different reconstruction points, which is a set of four in our case. These four reconstruction point values are compared with the received value, and the reconstruction point that returns least difference value, as shown in Equation (8) is considered the decoded message.

(8) $$m_{d}=\underset{\begin{array}{c} i\in 0,1 \end{array}}{\mathrm{argmin}}\left|s_{w}^{{}^{\prime }}\left(s_{i},m_{d}\right)-s_{w}\left(s_{i},m_{i}\right)\right|$$

where, $s_{w}^{{}^{\prime }}\left(s_{i},m_{d}\right)$ represents a distorted received signal, $m_{i}$ is the embedded message and $m_{d}$ is the decoded message. The decoding step also regenerates the embedded sequence following the same procedure explained in Section 4.1 as it receives the same GPS timestamp over CAN. Here it is assumed that the sensors are time-synchronized by a universal timestamp provider such as a GPS sensor [31].

## 5. Security Analysis and Performance Evaluation

Successful attacks are the ones that go undetected by the detection framework. In this section, we discuss various attack scenarios possible if an attacker gets access to the vehicle network and how the proposed framework can detect and localize these attacks. As a part of the attack model, we assume that the attacker has a good knowledge of the vehicle network protocols and automotive electrical system architecture. He has tools available to sniff the vehicle network and replay the modified messages on CAN/CAN-FD. With this knowledge, we identified three ways the attacker can modify the sensor data once he sniffs it from within the network. The attacks are be broadly classified as

- Data addition;
- Data deletion;
- Data modification.

For each of these attack scenarios in the following sections, we analyze how the proposed framework performs.

### 5.1. Data Addition

**Figure 9:**
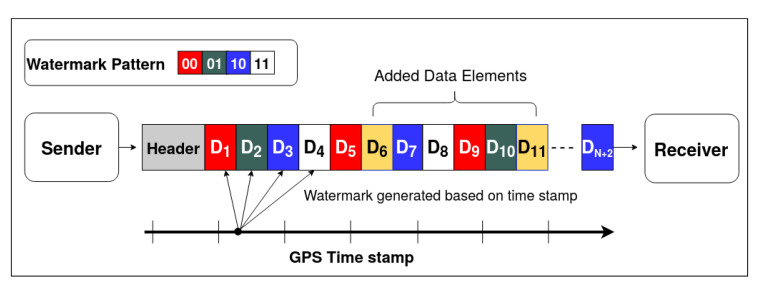
Data addition attack vector depiction.

**Figure 10:**
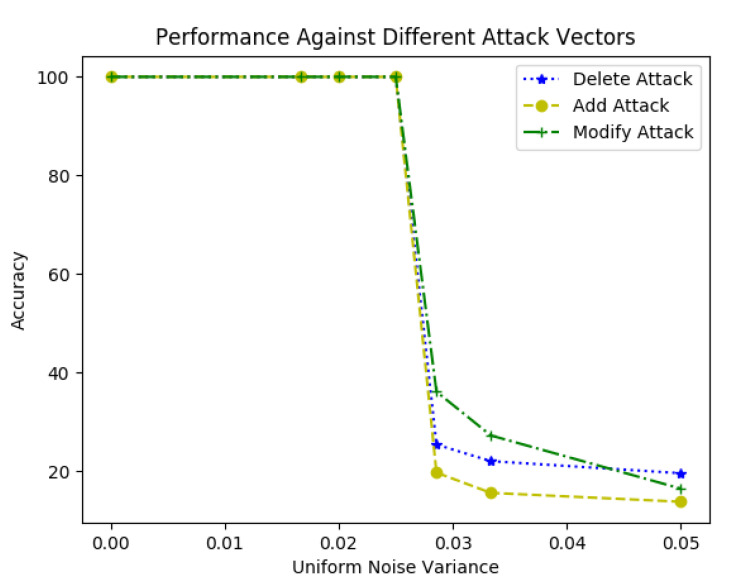
Tamper localization algorithm performance under varying channel noise.

Data addition is an attack scenario where the attacker modifies the radar reflections or tracklets with additional fake data elements either by copying the existing elements or by adding completely random data. A typical add attack scenario is represented in Figure 9, the data elements $D_{6}$ and $D_{11}$ are added to the original sequence of the radar data, increasing the total count of elements from *n* to n+2. With the proposed framework, position information (x,y) is 2D QIM encoded by the sender with a message pattern generated based on the GPS timestamp, as explained in Section 4.1. This message pattern is represented by the color-code (red, green, blue, and white) in Figure 9. Here, for simplicity, we assume a fixed pattern length of four but the framework can accommodate variable length patterns. With the additional data elements added during an attack, even if they are a copy of the existing data elements, the encoded sequence gets disrupted. The receiver expects a message sequence of green for data element $D_{6}$ and blue for data element $D_{11}$, but the algorithm detects the subsequent received elements do not have the expected message sequence. Here, we assume that the receiver knows the length of expected data elements. The sender and receiver can agree on a pre-defined range of values for the length or have an increment counter in each data-element, etc., to get the length. Knowing the encoded data element length along with the side information received from a different sensor modality, such as the GPS timestamp, as mentioned in Section 4.1, will help the decoder to generate the encoded message pattern. Based on the lengths of the encoded message sequence $l_{encode}$ and the decoded message sequence $l_{decode}$, the type of attack can be determined; i.e., it can be determined that the elements are added if $l_{decode}>l_{encode}$. As shown in Algorithm 2, the decoded message sequence is compared with the expected message sequence in an O(N) loop to find out the location of newly added data elements. This algorithm assumes that the added element’s pattern is different from its adjacent element. Figure 10 depicts the performance of the algorithm in the presence of additive uniform noise. The results show the robustness of the tamper detection algorithm and the proposed data hiding-based framework performance in the presence of channel noise. The QIM-based methods can recover the watermark as long as the channel noise is confined to the below equation.

(9) $$d_{min}^{2}>4\cdot N\cdot \sigma_{n}^{2}$$

where σ is the standard deviation of channel noise, *N* represents the number of encoding bits or dimensions, and $d_{min}$ represents the minimum distance between the reconstruction points [30]. It can be observed from Figure 10 that the proposed framework can detect and localize the tampered data elements with 100% accuracy when the noise is within bounds as per Equation (9), for a given step-size of Δ=1 cm.
**Algorithm 2:** Find added indices.
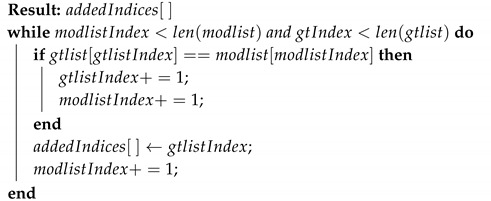


### 5.2. Data Deletion

**Figure 11:**
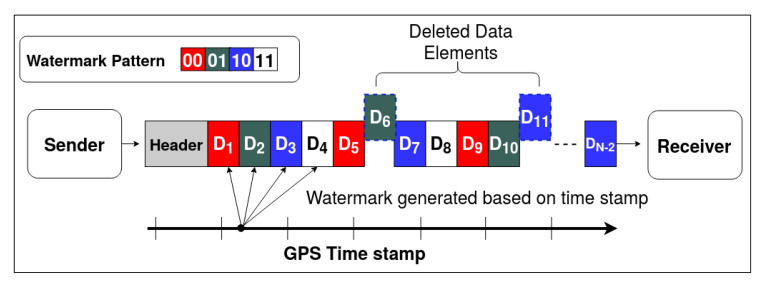
Data deletion attack vector depiction.

In this scenario, as shown in Figure 11, attacker modifies the radar detections either by carefully eliminating chosen targets or by deleting random elements. A typical delete attack scenario is represented in Figure 11, the data elements $D_{6}$ and $D_{11}$ are deleted from the original sequence of the radar data. This decreases the total count of elements from *n* to n−2. In the proposed method, the sender embeds the message pattern generated from GPS timestamp in the position information (x,y) of the data elements. The message pattern is represented by the color-code (red, green, blue, and white) in Figure 11. When the data elements get deleted, the message embedding sequence gets disrupted. The receiver expects a message sequence of green for data element $D_{6}$ and blue for data element $D_{11}$, but it can detect that the received elements $D_{7}$ and $D_{12}$ respectively do not have the expected pattern. Based on the lengths of the encoded message sequence $l_{encode}$ and the decoded message sequence $l_{decode}$, the type of attack can be determined; i.e., it can be determined that the elements are deleted if $l_{decode}<l_{encode}$.

The tamper localization algorithm is shown in Algorithm 3. The decoded message sequence is compared with the expected message sequence in an O(N) loop to determine the location of the deleted data elements. The results of the algorithm are shown in Figure 10. The algorithm detects and localizes the delete attack vector with 100% accuracy as long as the noise is bounded by Equation (9). It can be observed from Figure 10 that as the noise variance increases, the accuracy falls for a given step-size.
**Algorithm 3:** Find deleted indices.
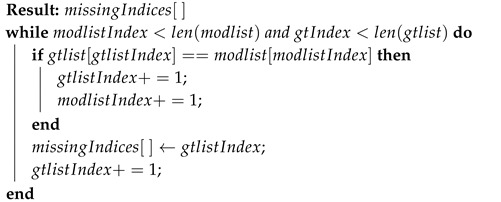


### 5.3. Data Modification

**Figure 12:**
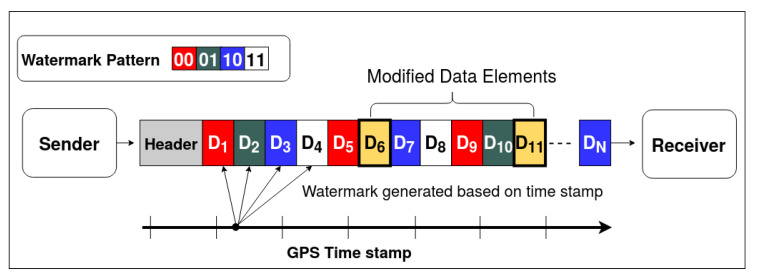
Data modification attack vector depiction.

During a data modification attack, as shown in Figure 12, the attacker modifies the radar detections by altering the existing data element content. Figure 12 represents a typical data modification attack. The data elements $D_{6}$ and $D_{11}$ are modified in the original sequence of the radar data. This type of attack does not change the total count of elements. In the proposed method, the sender embeds a message pattern generated from GPS timestamp, as explained in Section 4.1, into the position information (x,y) of the data elements. The message pattern is represented by color-code (red, green, blue, and white) in Figure 12. When the data elements get modified, the embedded message sequence gets disrupted. The receiver expects a message sequence of green for data element $D_{6}$ and blue for data element $D_{11}$ and detects that the received data elements do not have the expected pattern. To get the location of the modified data elements, as shown in Algorithm 4, the decoded message sequence is compared with the expected message sequence in an O(N) loop. The accuracy of the algorithm is represented in Figure 10. This algorithm assumes that random elements are modified within a given message pattern, and the channel noise is less than the step size. It is observed from Figure 10 that as the noise variance increases, the localization accuracy of the algorithm decreases. However, when the noise is within bounds, the detection and localization accuracy of the modified data elements is 100%—similar to the trend observed in the other two algorithms.
**Algorithm 4:** Find modified indices.
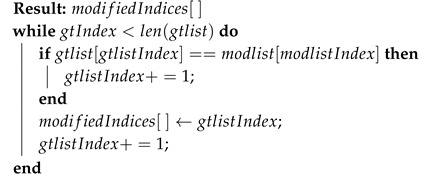


## 6. Experiments and Results

### 6.1. Impact of Embedding Distortion on Object Detection

Though, the data hiding-based sensor integrity framework is computationally less complex than the traditional cryptography, one of the major concerns with the method is the embedding-induced distortion as the watermark is embedded directly into the data by altering it. A question arises whether the end application or the consumer of this data can handle this distortion. An answer to this question would help the automotive industry adopt the watermarking techniques for sensor data or other data integrity verification applications. In this study, we analyze the effects of embedding radar data using the proposed 2D QIM data hiding framework on a sensor fusion algorithm. The sensor fusion method we use for this study is EKF. In autonomous vehicles, Kalman filters are used to estimate the state of any dynamic system, such as position estimation of moving objects on the road. In doing so, the Kalman filter only needs current observations and previous predictions, hence Kalman filter is a light-weight fusion algorithm [32,33,34]. They are also good at handling the measurement inaccuracies in the sensors, i.e., sensor noise. The EKF based sensor fusion algorithm takes inputs from two or more sensors and generates a combined prediction of the tracked object at every time step. The impact of the proposed 2D QIM method for sensor data integrity verification is estimated based on the output of the EKF. As explained in Section 3.1, in this experiment, we use measurements from two onboard vehicle sensors LiDAR and radar to estimate the state of a pedestrian moving in-front of the car. The same object, in this case a pedestrian, will be detected by the two sensors, and a Kalman filter fuses the data and predicts the accurate position of the pedestrian.

To predict the position of a target, a Kalman filter uses a motion or process model that estimates the future location of the target or object of interest. In this paper, we use a constant velocity motion model as a baseline for target motion estimation. The motion model, as depicted in Figure 4, predicts the position and velocity of the pedestrian at a future time $t_{k+1}$, based on the values at time-step $t_{k}$. This position information is provided as a 2D position and velocity vector called the state vector. In the context of this paper, the state vector consists of the predicted position and velocity of the pedestrian represented as

(10) $$x={\left[p_{x}\;p_{y}\;v_{x}\;v_{y}\right]}^{T}$$

where ($p_{x},p_{y})$ are (x,y) components of pedestrian position and ($v_{x},v_{y})$ are (x,y) components of his velocity at a given time step $t_{k}$. The Kalman filter consists of prediction and update steps. In the prediction step, the state vector $x^{{}^{\prime }}$ at next time step $t_{k}$ is estimated along with the uncertainty $P^{{}^{\prime }}$ based on values of *x* and *P* at previous time step $t_{k-1}$ and the motion model. During the update step, for every new measurement at time $t_{k}$, the estimation function performs the measurement update. The deterministic part of the prediction step *F* is the state transition matrix. The uncertainty measure *P* is a stochastic process modeled as random noise that affects the prediction step. The state vector $x^{{}^{\prime }}$ can be estimated as

(11) $$x^{{}^{\prime }}=f\left(x,\mu \right)$$

where,μisthestochasticpart,representedasN(0,Q), this can be re-written as

(12) $$\begin{array}{cc} \; & x^{{}^{\prime }}=Fx+\mu \\ \; & P^{{}^{\prime }}=FPF^{T}+Q \end{array}$$

where *F* is the state transition matrix that models state transitions from previous time step $t_{k-1}$ to current time $t_{k}$, μ is the added noise, *Q* is the process co-variance matrix that models the stochastic part of the state transition function. A linear motion model with constant velocity is used to define the state transition matrix *F*. The position at next time step $t_{k}$ is given by

(13) $$p_{t_{k}}^{{}^{\prime }}=p_{t_{k-1}}+v_{t_{k-1}}*\delta t$$

where $\delta t=t_{k}-t_{k-1}$ and since the model assumes constant velocity, the velocity at next time step is given as

(14) $$v_{t_{k}}^{{}^{\prime }}=v_{t_{k-1}}$$

based on the above model, the Kalman filter uses the estimated state to predict the pedestrian position.

In the update step, the sensor measurements are used to correct the predicted states and to obtain more accurate estimates. In the measurement function the vehicle only senses the pedestrian position and can be expressed as

(15) $$z={\left[p^{{}^{\prime }}\right]}^{T}$$

The measurement step that precedes the update step relies on the measurement model, measurement matrix *H*, and covariance matrix *R* to correctly estimate the measurement vector *z*. The measurement matrix is required to transform the measurement vector *z* to the state vector as shown in Equation (10). The measurement function can be represented as

(16) z=Hx+ω

where *H* is the measurement matrix that projects object position belief into the measurement space of the sensor and ω is the measurement error that encompasses all the uncertainties in measurements from the sensor represented as a Gaussian with zero mean and covariance matrix *R*, ω≈N(0,R). Assuming the measurement components are not cross-correlated, the covariance matrix *R* becomes a diagonal matrix. The dimensionality of *R* depends on the size of the measurement vector *z*, which is two for LiDAR and three for radar in our case. Hence, *R* becomes a 3×3 diagonal matrix for radar and a 2×2 diagonal matrix for LiDAR. The measurement matrix *H* also differs based on the sensors used by the fusion algorithm. Since LiDAR measures the position of the target in the Cartesian coordinates (x,y), the state vector to measurement vector transition is linear, and calculation of the measurement matrix *H* is straightforward. It just needs to discard the velocity from state vector. Hence during the update step, standard Kalman filter transitions are applied for LiDAR measurements. In the case of radar, the transition is non-linear as radar measures ρ, ϕ, $\dot{\rho }$ of the target. During the update step, to handle the non-linear measurement functions for radar measurements, we use EKF concept. Kalman filters are linear estimators, the extension of this idea to non-linear systems is called extended Kalman filter (EKF) [34]. In EKF, the non-linear state and observation equations are linearized using Jacobian matrices. Hence for radar sensor Jacobian of *H* is computed to get the linear approximation. Once the *z* is computed, the update step or correction step is performed where the latest measurements are used to update the state estimates and its uncertainties as follows:

(17) $$y=z-Hx^{{}^{\prime }}$$

Here *y* is the error value or the difference between the prediction and actual measurement at a given time step. The estimation error *S* is computed as

(18) $$S=HP^{{}^{\prime }}H^{T}+R$$

The Kalman gain *K* is computed as

(19) $$K=P^{{}^{\prime }}H^{T}S^{-1}$$

After the computation of the Kalman gain, the predictions are updated using the following equations and these steps are repeated for the entire drive cycle.

(20) $$x=x^{{}^{\prime }}+Ky$$

(21) $$P=\left(I-KH\right)P^{{}^{\prime }}$$

**Figure 13:**
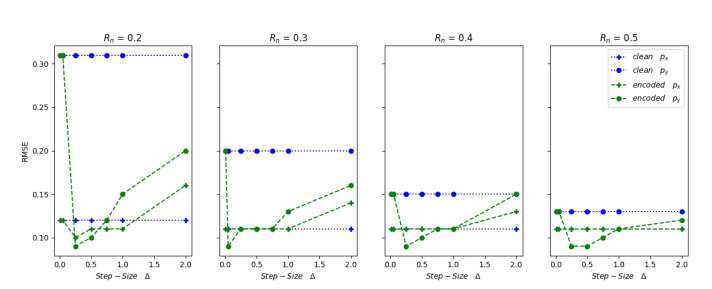
RMSE comparison at $R_{m}=0.4$.

**Figure 14:**
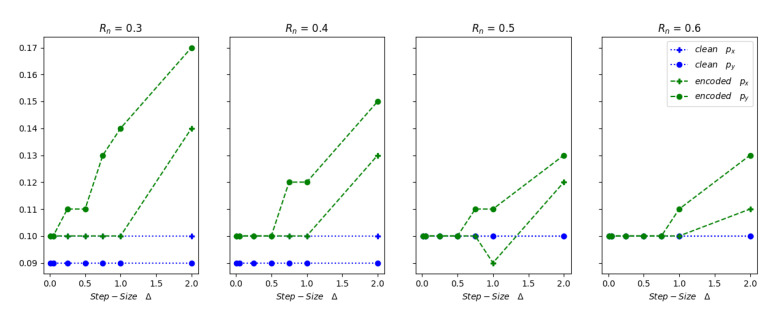
RMSE comparison at $R_{m}=0.5$.

It can be observed that in an EKF, the uncertainty in both the process and measurements are taken into consideration. In general, the measurement uncertainty or the measurement noise covariance matrix *R* in Equation (16), is the inherent sensor behavior and hence provided by the sensor manufacturer. Whereas, the process uncertainty *Q* in Equation (12), is defined based on the motion model and other application related assumptions. If we use the EKF as the sensor fusion algorithm, three configuration parameters can affect the algorithm outcome in the proposed framework. The first being the measurement noise matrix *R*, second the overall process noise *Q*, and third, the embedding step-size Δ. In this experiment, we analyze the impact of the watermark embedding using the 2D QIM method on the sensor fusion algorithm’s output under different configuration scenarios. We use two types of radar data as an input to the EKF algorithm that predicts the state vector of the pedestrian. The first type is the clean and unmodified data, and the second type is 2D QIM modified data. The resulting predictions from EKF are compared against the ground truth position vectors in both the cases using root mean square error (RMSE) metric calculated as follows:

(22) $$RMSE=\sqrt{\frac{1}{n}\sum_{t=1}^{n}{\left(x_{t}^{gt}-x_{t}^{pred}\right)}^{2}}$$

where $x_{t}^{gt}$ and $x_{t}^{pred}$ are the ground truth and predicted position vectors respectively at a given time *t* and *n* is the length of data. The RMSE value is used to determine the accuracy of the prediction. Low RMSE value indicates that the sensor fusion algorithm predicted the tracked object position accurately throughout the track path. The RMSE values of position vector $\left(p_{x},p_{y}\right)$ predictions generated from clean and watermarked inputs to the EKF are shown in Figure 13 and Figure 14.

The measurement input to EKF has a measurement noise component which is dependent on the intrinsic electronic characteristics of the sensor. This can be represented as an additive Gaussian noise ω as shown in Equation (16). The measurement noise covariance *R* represents the deviation of the sensor measured values form the true values. This deviation is estimated during the calibration phase by the sensor manufacturer. In the absence of the sensor manufacturer data, it can also be estimated using analytical methods [35]. To accurately compensate for the measurement noise in an EKF the *R* value for a given sensor must be known or estimated to be used in Equation (16).

If we consider $\omega_{R_{m}}\approx N\left(0,R_{m}\right)$ as the known or measured measurement uncertainty and $\omega_{R_{n}}\approx N\left(0,R_{n}\right)$ as the overall measurement uncertainty used in the sensor fusion EKF algorithm in Equation (16), an EKF provides accurate predictions when the value of $R_{n}\geq R_{m}$. Here, it is always better to keep the $R_{n}$ and $R_{m}$ values close to each other. If the EKF requires an inflated $R_{n}$ value to incur correct predictions, then it could be concealing other issues in the measurements, such as measurement outliers and non-Gaussian nature of the noise. The measurement uncertainty values used in the EKF $\omega_{R_{n}}$ can be represented as a combination of two or more different noise distributions with data satisfying the i.i.d. criteria. Let us say $\omega_{R_{n}^{1}}\approx N\left(0,R_{n}^{1}\right)\;\mathrm{and}\;\omega_{R_{n}^{2}}\approx N\left(0,R_{n}^{2}\right)$ are two different noise distributions that contributed to the overall noise $\omega_{R_{n}}$; then the resulting distribution can be represented as:

(23) $$\omega_{R_{n}}=N\left(0,R_{n}^{1}+R_{n}^{2}\right)$$

The RMSE results depicted in Figure 13 and Figure 14 show that the 2D QIM embedded radar data can be considered as an added random noise contributor to the overall measurement uncertainty and it can be represented by $R_{n}^{1}$ or $R_{n}^{2}$ in Equation (23). In this experiment, the RMSE values for clean and 2D QIM embedded radar data are calculated at different measurement noise covariance matrix values $R_{m}\in \left(0.4,0.5\right)$, $R_{n}\in \left(0.2,0.3,0.4,0.5,0.6\right)$, and varying embedded step sizes Δ∈(0.01,0.05,0.25,0.50,0.75,1,2) m. Considering the $R_{m}$ as the measurement error covariance provided by the sensor manufacturer, the EKF which accepts this radar sensor data should use a covariance matrix value $R_{n}$ above or equal to the $R_{m}$ uncertainty. It can be observed from Figure 13, when $R_{n}\geq R_{m}$, the RMSE values of position vector for the 2D QIM embedded data are less than or equal to the RMSE values from clean data for step-size Δ<0.75 m. With a given range of $p_{x}\approx 18.5$ m and $p_{y}\approx 12.5$ m in the data under test, the results show that the fusion algorithm can recover from position data perturbations of up-to 6%. As the $R_{n}$ value goes below the $R_{m}$, the RMSE of encoded data is less than the clean data only when Δ<0.05 m. This shows that the embedding-induced distortion at higher step sizes acts like additional uncompensated noise and introduces prediction errors—similar results are observed for the state vector predictions in case of data with measurement covariance matrix value $R_{m}=0.5$, as shown in Figure 14. It can be inferred from the results that in the case where the measurement covariance $R_{n}<R_{m}$, as the embedding step-size increases, the measurement noise value increases and hence the predictions of the embedded data elements are off. However, as the $R_{n}$ value is increased above the $R_{m}$, the embedding-induced distortion is gracefully handled by the fusion algorithm, and we observe low RMSE values even at larger step sizes. This phenomenon can be explained by Equation (23). Here the embedded-induced distortion acts like additive Gaussian noise component. The inherent randomness in the watermark generation and embedding, which acts as noise, adds up to the randomness in the sensor noise. These two noises are independent of each other; hence the resultant effect is additive. This increases the RMSE value of the prediction error when the fusion algorithm does not consider and compensate for this additional noise. These experiments, when repeated at different permissible values of process noise covariance matrix Q>0, showed similar results.

Apart from the embedding-induced distortion analysis, two different experiments are conducted to measure the other performance parameters of the detection framework, such as the bit error rate and the false alarm rate.

### 6.2. Bit Error Rate

In this experiment we analyze the errors in the decoded bit stream in the presence of channel noise. The decoder step in the proposed framework generates a binary message stream $M_{x,y}=\left\{m_{x,y}^{1},m_{x,y}^{2},\cdots ,m_{x,y}^{N}\right\}$, from the radar data elements. The bit error rate BER is calculated by comparing each bit in the decoded message $m_{x,y}^{i}\in \left\{m_{x}^{i},m_{y}^{i}\right\}$ with the embedded bit ${\hat{m}}_{x,y}^{i}$ as follows:

$$BER=\frac{\sum_{i=1}^{n}I_{m_{x,y}^{i}\neq \;{\hat{m}}_{x,y}^{i}}}{n}$$

where I is the indicator function, and *n* is the size of the decoded message bitstream. When no additional noise is added to the radar data elements, the BER is close to 8.6%, which corresponds to the noise due to the attack vectors. As the channel noise modeled by an uniform distribution is added to the data, the BER stays below 9.5% for the noise variance σ<Δ/5.65, for a given step-size Δ. As the noise variance increases beyond the threshold in Equation (9), the BER value increases as shown in Table 1. It can be observed that the robustness to the channel noise is directly proportional to the step-size Δ, which-in turn is directly proportional to the embedding-induced distortion.

### 6.3. False-Alarm Rate Analysis

The false alarm rate analysis is another important performance indicator of the proposed framework. It measures the number of data-elements the framework determines as tampered when it is provided with clean or unmodified data. In this experiment, the framework is tested with a combination of clean and modified data elements and the false alarm rate $f_{AlarmRate}$ is calculated as follows:

$$f_{AlarmRate}=N_{FalsePositive}/N_{DataElements}$$

where, $N_{FalsePositive}$ is the number of data elements the framework falsely classified as tampered and $N_{DataElements}$ is the total number of data elements tested. The experiment is repeated at different levels of the additive uniform noise to replicate the channel noise. The results are shown in Table 1. It can be observed that the $f_{AlarmRate}$ stayed at 0% when the uniform noise variance $\sigma <d_{min}/\left(2*\sqrt{(}N\right))$, where $d_{min}=\Delta /2$, N=2 in our framework. As the noise variance increases beyond this threshold the false positives increase resulting in higher false alarm rate. It can be concluded from these results that when the channel noise is within acceptable bounds, our framework can achieve 100% detection accuracy with zero false positives.

## 7. Conclusions

**Figure 15:**
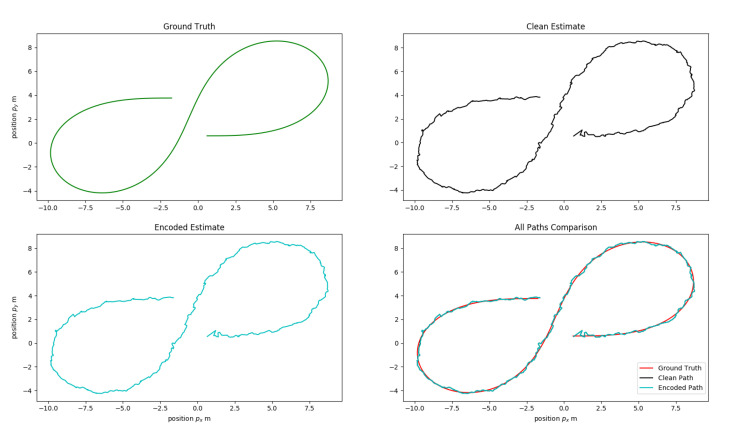
Comparison: EKF path prediction from clean and encoded data at $R_{m}$ = 0.5, $R_{n}$ = 0.5 and Δ = 0.01 m.

Cyber-physical system, such as an autonomous vehicle, is susceptible to insider attacks targeting sensors and their transmission channels, making it necessary to verify the integrity of sensor data before acting on it. Traditional data integrity protection methods, such as cryptography, cannot be applied in their entirety to solve this problem due to their resource requirements and complexity. In this paper, we propose a pipe-line based on watermarking to detect and localize tampering of the sensor data in an autonomous vehicle. This pipeline is tested for the affects of embedding-induced distortion using simulated radar data on an EKF based sensor fusion algorithm. The experimental results conclude that that the 2D QIM method for watermarking has a little or no effect on the EKF predictions for small values of quantization step-size Δ≤0.05 m, which can be attributed to the minimal distortion induced by the 2D QIM process. A visual representation of the tracked path by sensor fusion EKF algorithm for both plain and encoded inputs at a small step-size Δ=0.01 m is displayed in Figure 15. It can be observed that the predicted state-vectors for both the plain and 2D QIM embedded inputs are similar even when the actual measurement noise covariance $R_{m}$ and the EKF considered noise covariance $R_{n}$ are same. As the step size increases, the overall measurement noise covariance used in the EKF, $R_{n}$ need to take into account the noise generated by the 2D QIM embedding to get accurate results, this phenomenon is shown in Figure 13 and Figure 14. Other experiments to measure the tamper localization accuracy and noise resilience of the proposed framework show that the proposed framework works well if the channel noise of the interface is within theoretical bounds presented in Equation (9). The tamper localization accuracy of our framework is close to 100% when the interface noise is zero. In automotive networks, sensor data interfaces like CAN and Ethernet are wired, and the channel noise is minimal. Hence, using a 2D QIM approach with small step size can produce good tamper detection and localization accuracy and minimal data distortion.

In the security world, often times a layered architecture is preferred wherein if an attack cannot be prevented; It can be detected to prevent the worst outcome. We believe that watermarking the sensor data adds another layer to the security scheme using some light-weight and yet efficient techniques. These techniques can be used either in a standalone mode or in conjunction with traditional cryptography methods where-ever necessary, to secure data transfers over any physical interface such as CAN/CAN-FD, Ethernet, etc. In future, we plan to extend the proposed framework to different sensor modalities, different watermark embedding methods and intend to study the affects of embedding-induced distortion on more complex process models and state vectors.

## Figures and Tables

**Table 1:** BER and false-alarm rate at different noise levels.

Noise Variance σ		BER %	FalseAlarm %
0.0		8.6	0.0
Δ/6		9.2	0.0
Δ/5		9.2	0.0
Δ/4		8.6	0.0
Δ/3.5		18.6	61.1
Δ/3		28.6	75.0
Δ/2		56.4	85.2
